# A Multispecies Biofilm In Vitro Screening Model of Dental Caries for High-Throughput Susceptibility Testing

**DOI:** 10.3390/ht8020014

**Published:** 2019-05-30

**Authors:** Lara A. Heersema, Hugh D. C. Smyth

**Affiliations:** 1 Department of Biomedical Engineering, Cockrell School of Engineering, The University of Texas at Austin, Austin, TX 787812, USA; Lheersema@utexas.edu; 2 Division of Molecular Pharmaceutics and Drug Delivery, College of Pharmacy, The University of Texas at Austin, Austin, TX 78712, USA; 3 The LaMontagne Center for Infectious Disease, The University of Texas at Austin, Austin, TX 78712, USA

**Keywords:** multispecies biofilms, *Streptococcus gordonii*, *Streptococcus mutans*, *Candida albicans*, dental caries, high-throughput

## Abstract

There is a current need to develop and optimize new therapeutics for the treatment of dental caries, but these efforts are limited by the relatively low throughput of relevant in vitro models. The aim of this work was to bridge the 96-well microtiter plate system with a relevant multispecies dental caries model that could be reproducibly grown to allow for the high-throughput screening of anti-biofilm therapies. Various media and inoculum concentrations were assessed using metabolic activity, biomass, viability, and acidity assays to determine the optimal laboratory-controlled conditions for a multispecies biofilm composed of *Streptococcus gordonii*, *Streptococcus mutans*, and *Candida albicans*. The selected model encompasses several of the known fundamental characteristics of dental caries-associated biofilms. The 1:1 RPMI:TSBYE 0.6% media supported the viability and biomass production of mono- and multispecies biofilms best. Kinetic studies over 48 h in 1:1 RPMI:TSBYE 0.6% demonstrated a stable biofilm phase between 10 and 48 h for all mono- and multispecies biofilms. The 1:1:0.1 *S. gordonii*: *S. mutans*: *C. albicans* multispecies biofilm in 1:1 RPMI:TSBYE 0.6% is an excellent choice for a high-throughput multispecies model of dental caries. This high-throughput multispecies model can be used for screening novel therapies and for better understanding the treatment effects on biofilm interactions and stability.

## 1. Introduction

Dental caries, or tooth decay, is characterized by the acidic breakdown of dental hard tissues from the fermentation of dietary carbohydrates by oral microorganisms within biofilms on the tooth surface [1,2]. Of the over 600 species of bacteria, fungi, viruses, and archaea that have been identified in the human oral cavity using 16sRNA, pyrosequencing (ITS), and next-generation sequencing [3,4,5,6,7], an average of 100 microorganisms are harbored at any one time in an individual’s mouth [5] and 20–30 microorganisms are predominately found in dental caries-associated biofilms, commonly referred to as dental plaque [8]. Biofilms, or the community of microorganisms that form on biotic and abiotic substrates, including the tooth surface, occur naturally in the oral cavity and can even provide benefits to the host [8,9]. However, when pathogenic biofilm species are abundant, dental caries, gingivitis, or periodontitis can develop [1,3,10,11]. When dental caries remains untreated, the demineralization of tooth enamel leads to caries lesions, cavitation, and tooth loss [12]. As people live longer and retain their teeth longer, researchers warn that caries prevalence will increase [13]. Patient risk is not only a factor of age, socio-economic status, and access to dental care, but is often highly dependent on the patient and dental health regimen, which can make providing proper dental treatment options challenging and which emphasizes the need for new treatment options [2,14].

The noninvasive, pharmacological treatment of dental caries is limited and includes various fluorides (sodium, tin, potassium, acidulated phosphate, silver diamine, or amine), chlorhexidine, essential oils, cetylpyridinium chloride, metal salts, and antibiotics [15,16,17,18,19]. Many of these therapies are used both as treatment and as preventative measures and have overall been shown to reduce caries risk and improve caries maintenance [20]. However, despite the long term use of some of these treatments, caries prevalence globally increased by 45% between 1990 and 2015 [13]. The discovery of new biofilm treatments is generally a prolonged process that begins by demonstrating the anti-biofilm potential in vitro before testing the treatment with rigorous and costly in vivo studies, often including relevant animal models and clinical trials. Excellent reviews of existing in vitro oral caries biofilm models have been recently published; therefore, only a brief summary of model system limitations is provided here [21,22,23,24,25].

Mono-species biofilms, typically of *Streptococcus mutans*, are simple dental caries models that can be easily adapted to 96-well microtiter plate high-throughput model systems, as well as more complex systems that better reflect in vivo conditions [26,27,28]. However, mono-species biofilms fail to capture the complex synergistic and antagonistic relationships that exist in oral caries biofilms. Dual-species biofilms are also frequently used with 96-well microtiter plate systems. Examples of caries associated dual-species biofilms are *S. mitis* + *S. mutans* [29], *S. mitis* + *Aggregatibacter actinomycetemcomitans* [29], *S. gordonii* + *C. albicans* [30,31], and *S. mutans* + *C. albicans* [32]. However, multi-species biofilms comprised of more than two species are more challenging to develop in 96-well microtiter plates. In the last year, a three species caries model comprised of *Actinomyces naeslundii*, *A. odontolyticus*, and *S. mutans* was reported by Cieplik et al. [33,34], demonstrating the increased research attention of developing more complex microbial communities in 96-well microtiter plate systems. Microbial consortia of 2–12 microorganisms and microcosm biofilms, inoculated from plaque or saliva samples, have been developed for larger model batch and dynamic systems such as 24-well plates (including the Amsterdam Active Attachment Model and the Zurich Biofilm Model) [35,36,37,38,39,40,41,42], chemostats [43], the artificial mouth model [44,45,46], and flow cells [23,47]. These systems allow for the growth of more complex biofilms over longer time scales and on surfaces that represent the tooth better than polystyrene [21]. However, these systems are often expensive, have a higher variability between inoculations [48], and can only handle a small number of experimental conditions and replicates per run [24]. Therefore, these systems are not suitable for high-throughput screening studies.

The discovery and optimization of new therapies can be accelerated by bridging a high-throughput screening (HTS) system, based on the 96-well microtiter plates, with a relevant, reproducible multispecies dental caries model. HTS systems balance cost, quality, and quantity in an effort to develop a list of potential therapies, or “hits”, for further analysis. Ideally, HTS systems would have enough replicates per screened sample to adequately interpret the statistical significance and prevent false positive or negative results [49]. With oral caries models, a high-throughput adaptable system would also reproduce, as much as possible, the complex, multi-kingdom balance of microorganisms as found in vivo [7,50]. It is important to note that there is no standard, or “core”, microorganism composition acknowledged for dental caries studies [51]. However, it is generally accepted that the pathogenic biofilms associated with dental caries arise from the dysbiosis of the oral microbiome [8,52]. HTS models that contain microorganisms associated with healthy and pathogenic enamel biofilms would be best situated to understand if potential treatments can repair a healthy balance of microorganisms in the oral cavity. For instance, mutans streptococci and lactobacilli are recognized as pathogenic microorganisms in caries biofilms, while mitis and viridians streptococci are associated with commensal enamel biofilms [10,51]. Finally, for HTS studies to expedite the development of therapeutics, they must be adaptable to fast assays that correlate well with standard antimicrobial evaluation assays (e.g., colony forming unit enumeration) [53].

Recent work with a 3-species oral candidiasis model demonstrates the potential for developing multispecies biofilms in 96-well microtiter plates [54]. In this study, three microorganisms were also selected for the development of the novel biofilm. The selection of the microorganisms was based on studies of the flora found within oral caries biofilms, reports of in vitro compatibility, and the cariogenic potential [6,31,32,35,55,56,57]. *S. mutans* is the most notable bacteria within the dental caries-associated biofilm. In addition, microorganisms were selected to represent commensal oral biofilms, as well as oral microbiome dysbiosis that leads to candidiasis. Specifically, *Streptococcus mutans (S. mutans), Streptococcus gordonii (S. gordonii)*, and *Candida albicans (C. albicans)* were chosen.

*S. mutans* is the most notable bacteria within the dental caries-associated biofilm and is widely acknowledged as an important microorganism responsible for acid production and as a significant contributor to the synthesis of the biofilm extracellular matrix (ECM) [58,59]. Of particular importance, *S. mutans*, along with other streptococci, produce insoluble glucans which contribute to the structure of the ECM [60]. In addition, the exoenzyme gluocosyltransferase (Gtfs), which is released by *S. mutans,* can bind to the surface of other bacteria in the biofilm, causing them to produce glucans and support the biofilm structure [60]. This Gram-positive bacteria is also able to thrive in acidic environments by modulating sugar metabolic pathways, and it assists in the creation of microenvironments that favor aciduric species and the acidic breakdown of the tooth hard surfaces [61,62]. In a number of observed cases, a shift to higher proportions of *S. mutans* has been associated with dental caries [51,63]. *C. albicans* is the most common human fungal pathogen and is known to form biofilms with multiple bacteria species, including symbiotic relationships with *S. mutans* and *S. gordonii* that are elaborated in the discussion section [9,64]. *C. albicans* is polymorphic, transitioning from single-cell budding yeast to filamentous forms—pseudohyphae and true hyphae- as the disease states progress [65]. Within the oral cavity, *C. albicans* is found in commensal biofilms on the oral mucosa; however, it is also associated with dental caries biofilms and oral candidiasis [10]. *C. albicans* is an opportunistic pathogen, and while it is maintained in healthy individuals a reduction in other oral microorganisms can allow for an increase in the fungal burden, which can lead to candidiasis, or oral thrush [66,67]. Therefore, *C. albicans* is also an important metric for ensuring that fungal overgrowth does not occur when bacteria are downregulated by novel treatments [9,11,68]. *S. gordonii* is a Gram-positive bacterium in the streptococci group viridians and is considered a health-associated commensal oral bacterium essential for the production of protective tooth biofilms [63]. *S. gordonii* is an initial colonizer of the tooth pellicle, along with other non-mutans streptococci, and it facilitates co-adhesion and co-aggregation with other microorganisms [7]. *S. gordonii* is also a known producer of glucans, which are an important part of the EPS and contribute to the acidic microenvironment of pathogenic biofilms [31].

The aim of this work was to develop a HTS system that can adequately reflect the multi-species interactions in caries biofilms, while maintaining the simplicity needed to ensure reproducibility and efficiency in screening studies. A three species microcosm model in 96-well plates was selected for this work. While dual-species models containing *S. mutans* + *C. albicans* [32] and *S. gordonii* + *C. albicans* [30,31] have been previously reported for 96-well model systems, the three-species combination of these microorganisms has not. The development of this consortia model is presented here with a focus on the growth media selection and inoculum concentration, in order to determine the optimal conditions for maintaining the microorganism balance. HTS adaptable assays (i.e., metabolic activity and biomass) are compared with traditional viable colony forming unit counts. Additionally, an HTS adaptable method of determining the cariogenic potential by measuring the pH of the biofilm supernatant is presented. Finally, the mechanical properties of the developed model are characterized, as this provides insight into the structural stability of the formed biofilm and is more frequently being used in the evaluation of biofilm susceptibility to treatments that target the biofilm extracellular matrix.

## 2. Materials and Methods

### 2.1. Microorganisms and Growth Conditions

*Streptococcus gordonii* DL1.1, *Streptococcus mutans* UA159, and *Candida albicans* SC5314, kindly donated by Dr. Marvin Whiteley at Georgia Technical Institute, were maintained in frozen stocks at −80 °C. *S. gordonii* and *S. mutans* were sub-cultured overnight at 37 °C, 5% CO_2_ on Trypticase Soy Agar (BD, Franklin Lakes, NJ, USA) with 6% yeast extract (Sigma, St. Louis, MO, USA) [69,70]. *C. albicans* was sub-cultured aerobically at 37 °C on Sabouraud Modified Agar (BD, Franklin Lakes, NJ, USA) [71]. Planktonic cultures were grown in various media to determine the optimal growth media for all strains, as discussed in the following section. *S. gordonii* and *S. mutans* planktonic cultures were grown statically for 18 h at 37 °C with 5% CO_2_ [30]. *C. albicans* was grown planktonically in an orbital shaker at 160 rpm and 37 °C for 18 h [72] (MaxQ Mini 4450 Shaker, Thermo Fisher Scientific, Marietta, OH, USA).

### 2.2. Biofilm Development in Various Media

Planktonic cultures were harvested by centrifugation (2706 relative centrifugal force (RCF), 10 min, 25 °C), washed once with PBS and suspended in one of nine standard microbiological media selected for testing (Table S1). The media included bacteria promoting—Brain Heart Infusion (BHI) (BD, Franklin Lakes, NJ, USA), Todd Hewitt Broth (BD, Franklin Lakes, NJ, USA) with 0.3% yeast extract (THBYE 0.3%) [30], and Trypticase Soy Broth with 0.6% yeast extract (TSBYE 0.6%) [69]; fungi promoting—Yeast Nitrogen Base (BD, Franklin Lakes, NJ, USA) (YNB) [73] and Sabouraud Dextrose Broth (SDB) [74]; and non-specific media—RPMI 1640 (VWR Life Science, Radnor, PA, USA) [75], 1:1 *v*/*v* RPMI 1640:THBYE 0.3%, 1:1 *v*/*v* RPMI 1640:TSBYE 0.6%, and M9 minimal salts (BD, Franklin Lakes, NJ, USA) [76].

100 μL of prepared dilutions of mono-species (1 × 10^7^ cells/mL for *S. gordonii* and *S. mutans*, 1 × 10^6^ cells/mL for *C. albicans*) or mixed microorganisms (1:1:0.01, 1:1:0.1, and 1:1:1 ratios of *S. gordonii: S. mutans: C. albicans*) in each media were pipetted into the wells of a flat-bottom tissue-culture treated 96-well microtiter plate (Corning^®^ Incorporated, Corning, NY, USA). The plates were incubated statically for 24 h at 37 °C and 5% CO_2_. After incubation, the biofilm metabolic activity, crystal violet biomass, viability, and pH assays were performed, as described below. For PrestoBlue^®^ measurements, fresh media was added to the wells corresponding to the growth media.

### 2.3. Inoculum Composition

The planktonic cultures were prepared as described above in 1:1 *v*/*v* RPMI 1640:TSBYE 0.6% media. Inoculum concentrations of 1.5 × 10^6^–1.5 × 10^7^ cells/mL for *S. gordonii* and *S. mutans* or 1.25 × 10^5^–1.25 × 10^6^ cells/mL for *C. albicans* were prepared by dilution. cell concentration ranges were selected based on previously reported biofilm models [30,31,74]. The cells were plated in 96-well microtiter plates in a sequential order for both the dual- and tri- species models: *S. gordonii*, *S. mutans*, and *C. albicans*. In the dual-species models, *S. gordonii* was plated in increasing concentrations as the column number increased. *S. mutans*, in combination with *C. albicans*, was plated similarly; however, when in combination with *S. gordonii*, *S. mutans* was placed in a decreasing concentration as the column number increased. In all of the models, *C. albicans* was plated in decreasing concentrations as the column number increased. In the tri-species model, both *S. mutans* and *S. gordonii* were plated at the same concentration, increasing as the column number increased. All biofilms were grown for 24 h at 37 °C and 5% CO_2_. After 24 h, the biofilm growth and viability parameters were measured using PrestoBlue^®^, XTT cell proliferation, crystal violet biomass, and microbial enumeration assays.

### 2.4. Kinetic Studies

For the 48-h kinetic studies, planktonic cultures were prepared as described previously in 1:1 *v*/*v* RPMI 1640:TSBYE 0.6% media. 100 μL of prepared dilutions with mono-species (1 × 10^7^ cells/mL for *S. gordonii* and *S. mutans*, 1 × 10^6^ cells/mL for *C. albicans*) or mixed microorganisms (1:1:0.01, 1:1:0.1, and 1:1:1 ratios of *S. gordonii*: *S. mutans*: *C. albicans*) were plated in each well of 96 well microtiter plates. At each time point in the 48-h study, the supernatant was removed and transferred to a new 96 well plate. The biofilm viability was measured using a PrestoBlue assay, and the pH was measured using a BCG assay, as described previously. Following the viability measurements, the supernatant was removed and replaced with 200 μL sterile PBS. At the end of all time points, the biofilms were either stained with crystal violet for a biomass determination or sonicated and diluted for microbial enumeration assays.

### 2.5. Biofilm Metabolic Activity

The biofilm metabolism was primarily estimated by adding 10 μL of PrestoBlue^®^ Cell Viability Reagent (Invitrogen^TM^, Carlsbad, CA, USA) and 90 μL of fresh growth media to each well [30,77]. PrestoBlue is a resazurin-based metabolic assay in which the blue non-fluorescent and non-toxic resazurin is reduced to resorufin, a pink and fluorescent dye, by the oxidoreductases within metabolically active cells [78]. Resazurin assays are non-toxic to eukaryotic and prokaryotic cells, allowing for additional assays to be performed [77,79,80]. The plates were incubated in the dark at 37 °C for 20 min, and the fluorescence intensity (Ex/Em: 560/590 nm) was measured using a microplate reader (Infinite M200 microplate reader, Tecan, Tecan Sp, Inc., Männedorf, Switzerland). Relative fluorescence units (RFU) are reported for all measurements. A 2,3–Bis(2–methoxy–4–nitro–5–sulfophenyl)–2*H*–tetrazolium–5–carboxanilide (XTT) cell proliferation assay was also performed for the inoculum concentration study, as described in prior work [76]. Briefly, an XTT working solution in PBS was prepared by combining PBS, XTT stock solution (1 mg/mL in PBS), and menadione solution (0.07 mg/mL in PBS) in a 79:20:1 proportion. 200 μL of the XTT working solution was added to each well of the 96 well plate and incubated in the dark at 37 °C for 4 h. 100 μL of the solution was transferred to a new 96 well plate, and the absorbance (AU) of the XTT reagent was measured at 492 nm.

### 2.6. Biofilm Biomass

The biomass was determined using a crystal violet assay, as described in prior work [81]. Biofilms were fixed with 200 μL methanol for 30 min at 25 °C and allowed to dry. The biofilms were then stained with 200 μL of 0.01% crystal violet (CV) solution for 30 min at 25 °C without shaking. A 0.01% CV solution was used in place of the traditional 0.1% CV solution to ensure that the absorbance was properly measured for each well [82]. The wells were washed twice with 200 μL sterile DI H_2_O and air dried at 25 °C. Finally, 200 μL of 30% acetic acid solution was added to each well, and the absorbance (AU) was read at 570 nm after a thirty-minute static incubation time at 25 °C.

### 2.7. Microbial Enumeration

Viable microbial colony counts were determined based on colony forming units (CFU). The wells were filled with 200 μL sterile PBS and sonicated for 30 min to disperse bacteria within the biofilm. The dispersed biofilms were serially diluted, and 10 μL were spotted on trypticase soy agar with 0.6% yeast extract (TSAYE 0.6%) plates (*all* microorganisms) [70], trypticase soy-sucrose-bacitracin agar with 0.01% 2,3,5–triphenyltetrazolium chloride (TYS20B + 0.01% TTC) plates (*S. mutans* selective) [83,84], or Sabouraud Modified agar plates (*C. albicans* selective) [57,85]. Colony forming units per milliliter were quantified after an inverted incubation for 24 h at 37 °C in air (Sabouraud Modified plates) or 5% CO_2_ (TSAYE 0.6% and TYS20B + 0.01% TTC plates). The results were reported as CFU/mL for biofilm viability. The approximate *S. gordonii* counts in the multispecies biofilms were calculated by determining the ratio of the maximum counts of *S. mutans* to *S. gordonii* grown independently on TSAYE 0.6% agar. This system of three agar plate types allowed for the detection of each microorganism species within the multispecies biofilms and the respective mono-species biofilms.

### 2.8. Quantification of Biofilm Supernatant pH

The high-throughput microenvironment acidity was determined by pipetting 100 μL of supernatant of the grown biofilms into new 96 well microtiter plates. 20 μL of 0.1% Bromocresol Green (BCG) was added to each well (Ricca Chemical Company, Arlington, TX, USA). Bromocresol dyes are useful pH sensitive dyes and have been used in agar selective media [86,87] and pH indicating media [88] for various microorganisms. The absorbance was read at the isosbestic point wavelength (512 nm) and the maximum absorbance wavelength (620 nm) after a 20-min incubation period. The absorbance ratio was calculated by dividing the maximum absorbance by the absorbance at the isosbestic point [89,90]. This ratio was compared to a standard curve of pH measured using a temperature compensated pH meter (Orion 350, Thermo Scientific, Marietta, OH, USA), with 12 mL biofilm suspensions to determine the acidity of the biofilm microenvironment (Figure S1).

### 2.9. Bulk Rheology

For the rheological studies, 250 μL of prepared dilutions with mono-species (1 × 10^7^ cells/mL for *S. gordonii* and *S. mutans*, 1 × 10^6^ cells/mL for *C. albicans*) or mixed microorganisms (1:1:0.01, 1:1:0.1, and 1:1:1 ratios of *S. gordonii*: *S. mutans*: *C. albicans*) were spread on Trypticase Soy Agar with 0.6% Yeast Extract (TSAYE 0.6%) plates and incubated at 37 °C and 5% CO_2_ for 24 h. For measuring mechanical properties, the biofilm was gently scraped directly onto a strain-controlled TA instruments AR2000ex rheometer (TA Instruments, New Castle, DE, USA), and the 8 mm plate rheometer geometry was lowered to a gap height of 500 μm [91]. The *S. gordonii* and *S. mutans* mono-species biofilms required three plates per measurement to ensure enough biofilm for accurate measurements. Excess biofilm was removed from around the rheometer geometry prior to measurements. A 3D printed acrylonitrile butadiene styrene (ABS) solvent trap lined with moist cotton balls was placed around the base of the rheometer and geometry to prevent biofilm drying during the measurements. Oscillatory frequency sweeps for 0.1 to 200 rad/s at 1% strain and oscillatory strain sweeps from 0.1 to 200% strain at 3.142 rad/s were performed at 25 °C on each sample each day. The plateau elastic modulus, G’, was taken as the elastic modulus of the linear region or approximately 1% strain. The yield strain, e_Y_, was determined from the intersection of the linear region and power region of the strain-sweep data. Finally, the yield stress, s_Y_, was determined based on the stress corresponding to the x-intercept of the intersection of the yield strain linear and the power regions. The representative frequency and strain sweep curves are shown in Figure S2.

### 2.10. Statistical Approaches

All studies were conducted with a minimum of three biological replicates. To ensure model reproducibility, independent trials were conducted over 6 months for the biofilm development in various media (3 replicates: Table S2), the 48-h kinetic growth study (2 replicates: Table S3), and the bulk rheology measurements (2 replicates). The replicate data is presented in the supplementary Tables S2 and S3. The error bars in all figures are standard deviations. The data were primarily analyzed using R open-source software [92]. The *p*-values were calculated using Tukey’s HSD (honest significant difference) test. The microbial enumeration results were analyzed using a two-tailed student’s T-test in Excel. The *p*-values indicated on the figures are as follows: *p* < 0.001 (***), *p* < 0.01 (**), *p* < 0.05 (*), and *p* < 0.1 (.).

## 3. Results

### 3.1. The Effect of Microbiological Media on Mono- and Multispecies Growth over 24 hours

#### 3.1.1. Biofilm Metabolism (PrestoBlue Assay)

**Figure 1:**
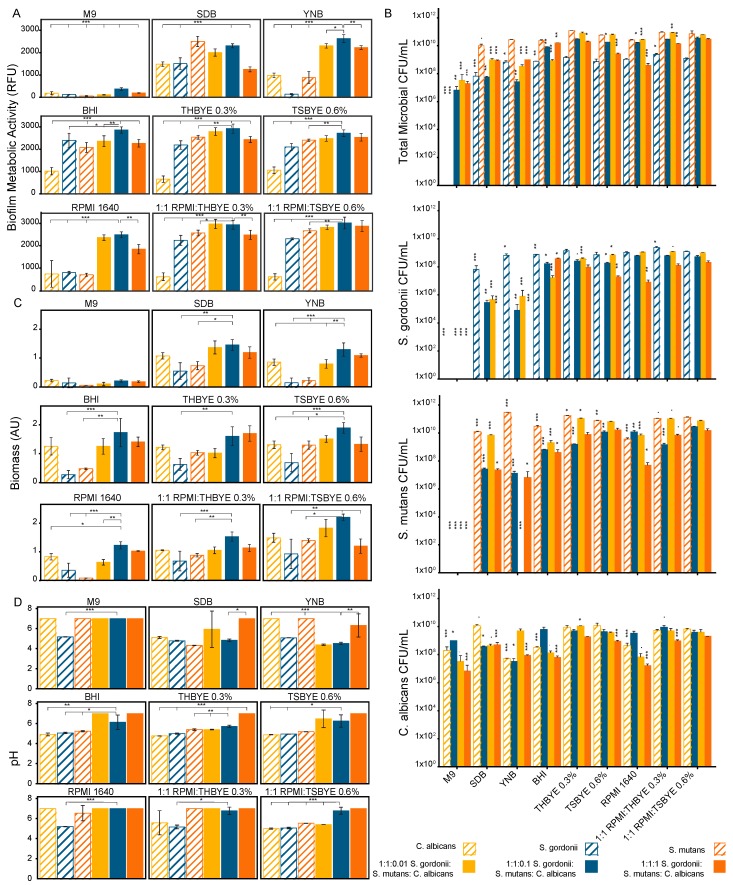
Mono-species and multispecies biofilm growth in different media assessed at 24 h, by (**A**) metabolic activity assay, (**B**) viability, (**C**) biomass, and (**D**) cariogenic potential assay. The biofilms were grown in M9, SDB, YNB, BHI, THBYE 0.3%, TSBYE 0.6%, RPMI 1640, 1:1 *v*/*v* RPMI 1640:THBYE 0.3%, and 1:1 *v*/*v* RPMI 1640:TSBYE 0.6% in 96-well microtiter plates for 24 h. The metabolic activity was measured by PrestoBlue^®^ fluorescence (RFU, n = 6). The viability was determined using colony forming units per milliliter (CFU/mL, n = 3). The total microbial counts and *S. gordonii* counts were performed using TSAYE 0.6% plates, the *S. mutans* counts were performed using TYS20B + 0.01% TTC agar plates, and the *C. albicans* counts were performed using Sabouraud Modified agar plates. The biofilm biomass was measured using crystal violet staining absorbance (AU, n = 3). The biofilm cariogenic potential was determined using Bromocresol Green pH sensitive dye absorbance (n = 6). For parts A, C, and D, the statistical significance was compared to the 1:1:0.1 biofilm growth in each media type, with *p*-values < 0.001 (***), 0.01 (**), or 0.05 (*). For part B, the significance was compared to the microorganism growth in 1:1 *v*/*v* RPMI 1640:TSBYE 0.6% media for each biofilm type. All error bars are the standard deviation.

We tested nine microbiological media including bacteria promoting (Brain Heart Infusion, Todd Hewitt Broth with 0.3% yeast extract, and Trypticase Soy Broth with 0.6% yeast extract), fungal promoting media (Yeast Nitrogen Base and Sabouraud Dextrose Broth), and non-specific media (RPMI 1640, 1:1 *v*/*v* RPMI 1640:THBYE 0.3%, 1:1 *v*/*v* RPMI 1640:TSBYE 0.6%, and M9 minimal salts). The biofilm growth and metabolic activity at 24 h, measured using a PrestoBlue viability assay, was dependent on the microbial composition of the biofilm and the media (Figure 1A). In 7 of the 9 media tested, the 1:1:0.1 multispecies biofilm was the most metabolically active of all the biofilm compositions that were tested. Statistical comparisons between the biofilms for each media type were carried out, with comparisons to the 1:1:0.1 multispecies biofilm used as a reference, as shown in Figure 1. In all except SDB, the 1:1:0.1 multispecies biofilm viability was significantly greater (*p* < 0.05) than all the mono-species biofilms. There was no statistically significant difference between *S. mutans* and the 1:1:0.1 multispecies biofilm in SDB. The mono-species biofilm growths were best supported by BHI, 1:1 *v*/*v* RPMI 1640:TSBYE 0.6%, and SDB for *S. gordonii*, *S. mutans*, and *C. albicans*, respectively. 1:1 *v*/*v* RPMI 1640:THBYE 0.3% supported the 1:1:0.01 multispecies biofilm and 1:1 *v*/*v* RPMI 1640:TSBYE 0.6% supported both the 1:1:0.1 and 1:1:1 multispecies biofilms best. The 1:1:0.1 multispecies biofilm had a significantly greater growth than the 1:1:1 multispecies biofilm in 7 of the 9 tested media and a significantly greater growth than the 1:1:0.01 multispecies biofilm in 3 of the 9 tested media.

#### 3.1.2. Biofilm Viability (Microbial Enumeration Assay)

Similar trends were seen between the PrestoBlue^®^ and CFU results. The colony enumeration was conducted for both the initial inoculum in each media type and again after 24 h of biofilm growth. The number of colony forming units per mL for each microorganism, as well as a total microbial count after 24 h of growth, are shown in Figure 1B. The total microbial count based on the colonies formed on TSAYE 0.6% agar, which supported all microorganisms in the multispecies biofilms, confirmed the media types that best supported the biofilm metabolic activity for the multispecies biofilms. For the mono-species biofilms, a deviation was seen from the biofilm metabolic data measured using PrestoBlue. The mono-species biofilm viability was best supported by THBYE 0.3%, YNB, and TSBYE 0.6% for *S. gordonii* (on TSAYE 0.6%), *S. mutans* (on TYS20B + 0.1%TTC), and *C. albicans* (on Sabouraud Modified), respectively. In addition, M9 media supported colony formation after 24 h for *C. albicans*, which was not indicated by the metabolic activity assay. It was also notable that the *S. gordonii* colony forming unit counts, or viable counts, were approximately two orders of magnitude lower than the *S. mutans* viable counts. This effect is more pronounced than the difference in the metabolic activity for the mono-species biofilms of *S. gordonii* and *S. mutans* but is also noted in the PrestoBlue assay for most of the tested media types.

#### 3.1.3. Biofilm Biomass (Crystal Violet Assay)

The biofilm biomass was more uniform across the media types than the viability was, with a stronger dependence on the *C. albicans* concentration (Figure 1C). The 1:1:0.1 multispecies biofilm produced the most biomass in all media except the THBYE 0.3% media. The 1:1:0.1 multispecies biofilm produced significantly more biomass than the *S. gordonii* or *S. mutans* mono-species biofilms in 7 of the 9 tested media compared to only 3 of the tested media for the *C. albicans* mono-species biofilm (*p* < 0.05). The 1:1:0.1 multispecies biofilm produced significantly more biomass than the other multispecies biofilms in 3 of the tested media types (*p* < 0.05).

#### 3.1.4. Biofilm Cariogenic Potential (pH Assay)

The biofilm supernatant pH was estimated using a BCG assay. In most of the tested media, the mono-species biofilms had lower supernatant pH values after 24 h of growth than the 1:1:0.1 multispecies biofilm did (Figure 1D). In all media, the 1:1:1 multispecies biofilm maintained a pH above 6. All other biofilms had pH levels below 6 depending on the media type.

### 3.2. The Effect of Inoculum Concentration on Dual- and Multispecies Biofilm Growth over 24 hours

#### 3.2.1. Biofilm Metabolism (PrestoBlue Assay)

**Figure 2:**
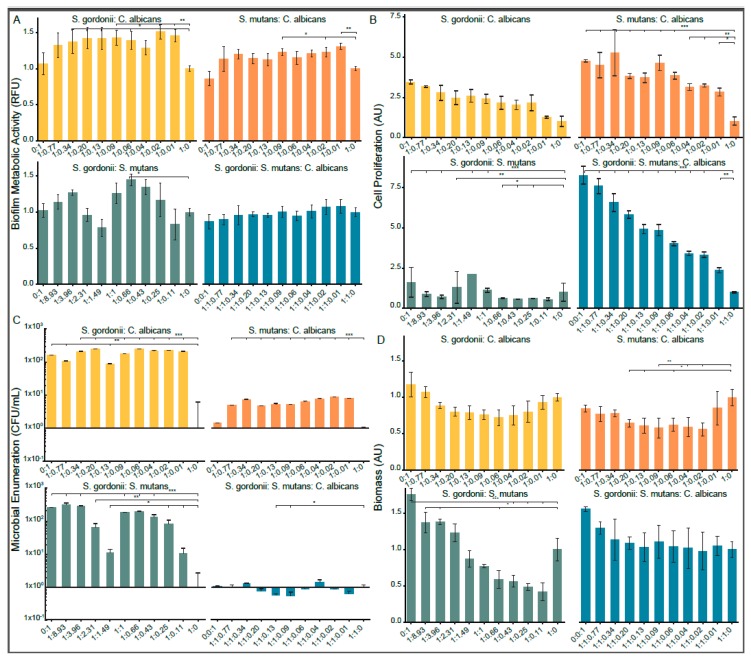
Assessment of the inoculum concentrations on the biofilm growth in 1:1 *v*/*v* RPMI 1640:TSBYE 0.6% media at 24 hours by (**A**,**B**) metabolic activity assays, (**C**) viability, and (**D**) biomass. The inoculum concentrations are expressed as ratios of Streptococci: Candida, *S. gordonii: S. mutans*, or *S. gordonii: S. mutans: C. albicans.* All data are normalized to the 1:0 or 1:1:0 ratios. The metabolic activity was measured either by (A) PrestoBlue^®^ fluorescence (RFU) or (B) XTT reduction absorbance (AU). The viability was determined using colony forming units per milliliter (CFU/mL). The total microbial counts on TSAYE 0.6% plates are shown. The biofilm biomass was measured using crystal violet staining absorbance (AU). The statistical significance is compared to the 1:0 or 1:1:0 ratios, with *p*-values < 0.001 (***), 0.01 (**), or 0.05 (*). All error bars are the standard deviation (n = 3).

After 24 h of biofilm growth, both the *S. gordonii*: *C. albicans* and *S. mutans*: *C. albicans* dual species biofilms were observed to have an increased cell metabolic activity compared to the mono-species biofilms (Figure 2A). The *S. gordonii*: *S. mutans* biofilm metabolic activity was concentration dependent. The tri-species mixed biofilm model metabolic activity was stable across all the tested inoculum concentrations with a slight increase for lower concentrations of *C. albicans*.

It was found that the XTT absorbance measurements were strongly dependent on the concentration of *C. albicans* for both the dual and tri species models (Figure 2B). However, for the *S. gordonii* and *S. mutans* dual biofilm, the XTT signal was very low at all concentrations. Due to the strong dependence on the *C. albicans* concentration and the inconsistency with other data, the XTT assay was deemed unacceptable for accurately determining the biofilm viability with the *S. gordonii*, *S. mutans*, and multispecies biofilms [30,71,93]. The inoculum concentrations are presented as ratios to the *S. gordonii* inoculum concentration (or *S. mutans* for the *S. mutans*: *C. albicans* dual species biofilm).

#### 3.2.2. Biofilm Viability (Microbial Enumeration Assay)

The total microbial counts based on the colony formation on TSAYE 0.6% agar plates were determined for all biofilms with patterns similar to the cell metabolic activity results from the PrestoBlue^®^ assay (Figure 2C). For both the *S. gordonii*: *C. albicans* and *S. mutans*: *C. albicans* dual species biofilms, the CFU counts were significantly higher for the dual species biofilms compared to the streptococci mono-species biofilms (*p* < 0.01). For the *S. gordonii*: *C. albicans* dual species biofilm, the *C. albicans* mono-species biofilm had significantly more colonies than the *S. gordonii* mono-species biofilm (*p* < 0.01). All dual species biofilms, as well as the *S. mutans* mono-species biofilm, formed significantly more colonies than the *S. gordonii* biofilm (*p* < 0.05). Only two inoculum ratios for the tri-species biofilm model formed fewer colonies than the Streptococci control biofilm.

#### 3.2.3. Biofilm Biomass (Crystal Violet Assay)

The crystal violet staining indicated a decrease in the biomass for all dual species models compared to the mono-species biofilms (Figure 2D). This reduction was most notable for the *S. gordonii*: *S. mutans* biofilm, where the introduction of *S. gordonii* decreased the biomass production. It was also noted from the crystal violet staining that each species within the biofilm produced different amounts of biomass when grown in a mono-species biofilm, with *C. albicans* > *S. mutans* > *S. gordonii* producing biomass.

### 3.3. Biofilm Growth Kinetics for Mono- and Multispecies Biofilms over 48 hours

#### 3.3.1. Biofilm Metabolism (PrestoBlue Assay)

**Figure 3:**
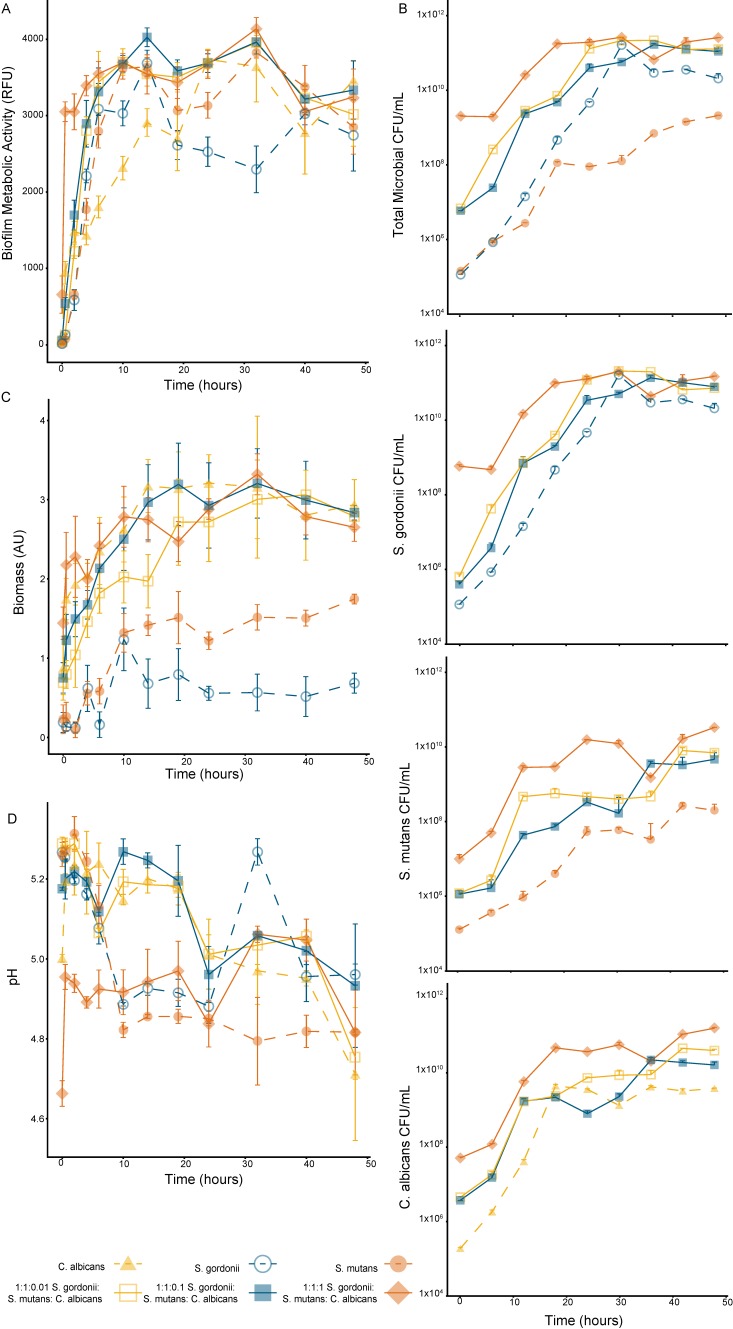
Assessment of the mono- and multispecies biofilm growth over 48 hours in 1:1 *v*/*v* RPMI 1640:TSBYE 0.6% media by (**A**) metabolic activity assay, (**B**) viability, (**C**) biomass, and (**D**) cariogenic potential assay. The metabolic activity was measured by PrestoBlue^®^ fluorescence (RFU, n = 6). The viability was determined using colony forming units per milliliter (CFU/mL, n = 3). The total microbial counts and *S. gordonii* counts were performed using TSAYE 0.6% plates, the *S. mutans* counts were performed using TYS20B + 0.01% TTC agar plates, and the *C. albicans* counts were performed using Sabouraud Modified agar plates. The biofilm biomass was measured using crystal violet staining absorbance (AU, n = 3). The biofilm cariogenic potential was determined using Bromocresol Green pH sensitive dye absorbance (n = 6). All error bars are the standard deviation.

The growth kinetics were tracked over a 48-h period for both the mono-species and multispecies biofilms, with a media replacement at the 24-h time point (Figure 3A). After 2 h (*p* < 0.01), all tested biofilm compositions had a metabolic activity significantly greater than the one at time zero, with the *C. albicans* mono-species biofilm and the 1:1:1 multispecies biofilm having significantly more metabolic activity after 30 min (*p* < 0.001) (Significance not indicated on the Figure 3). All multispecies biofilms’ metabolic activity was less variable after 10 h of growth compared to the mono-species biofilms. At 48 h, there was no significant difference in the metabolic activity between the 1:1:0.1 multispecies biofilm and any other tested biofilm composition.

#### 3.3.2. Biofilm Viability (Microbial Enumeration Assay)

The total microbial counts had similar trends to the cell metabolic activity data but with less fluctuation between the time points (Figure 3B). The total microbial count, based on the colonies formed on TSAYE 0.6% agar, showed a stable biofilm viability for all the multispecies biofilms after 19 h. For the mono-species biofilms, a stable biofilm viability was observed after 24 h, 19 h, and 14 h for *S. gordonii* (on TSAYE 0.6%), *S. mutans* (on TYS20B + 0.1%TTC), and *C. albicans* (on Sabouraud Modified), respectively.

#### 3.3.3. Biofilm Biomass (Crystal Violet Assay)

The biofilm biomass had a strong dependence on the *C. albicans* concentration (Figure 3C). After half an hour of growth, the *C. albicans* mono-species biofilm and all the multispecies biofilms had a significantly higher biomass production than the mono-species Streptococci biofilms (*p* < 0.05). As noted previously, the *S. mutans* mono-species biofilm produced more biomass than the *S. gordonii* mono-species biofilm.

#### 3.3.4. Biofilm Cariogenic Potential (pH Assay)

The biofilm pH declined over the 48-h growth period, except for the 1:1:1 multispecies biofilm, which had the most interference in the pH absorbance measurement due to the high concentration of *C. albicans* (Figure 3D). The *S. mutans* and 1:1:0.01 multispecies biofilms had the largest decrease in supernatant pH during the measurement period. All biofilms had a measured pH below 5 at 48 h.

### 3.4. Biofilm Structural and Mechanical Properties

**Figure 4:**
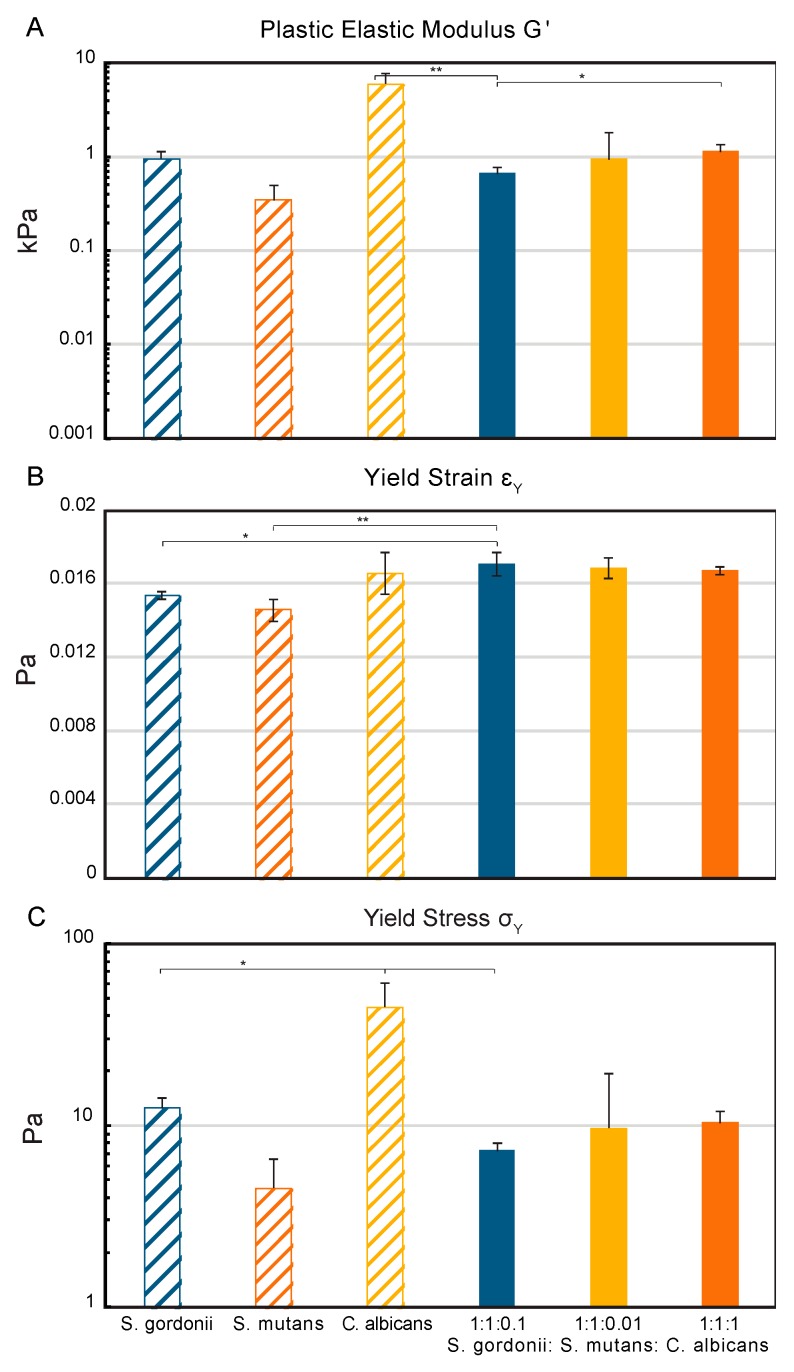
Structural characterization of the mono- and multispecies biofilms by the assessment of mechanical properties. The mechanical properties were measured using an 8 mm parallel plate rheometer at 25 °C. (**A**) The plastic elastic modulus G’ measures the rigidity of the biofilm; (**B**) The yield strain, ε_γ_, measures the deformation that can occur before mechanical failure begins; (**C**) The yield stress, σ_γ_, indicates the strength of the biofilm. The statistical significance is compared to the 1:1:0.1 biofilm, with *p*-values < 0.001 (***), 0.01 (**), or 0.05 (*). All error bars are the standard deviation (n = 3).

The macro-rheology studies, using oscillatory frequency and strain sweeps at 25 °C, revealed different viscoelastic properties between the three mono-species biofilms and the multispecies biofilms (Figure S2). The elastic modulus, G’, of the mono-species biofilms indicated that the *S. gordonii* and *S. mutans* biofilms are less rigid (0.3 and 1 kPa) than the *C. albicans* biofilms (6 kPa). Overall, the multispecies biofilms had an increasing elastic modulus as the concentration of *C. albicans* in the initial inoculum increased (0.6–1.1 kPa). The 1:1:0.1 multispecies biofilm had the lowest elastic modulus of the multispecies biofilms (0.6 kPa) and was significantly lower than both the *C. albicans* mono-species biofilm and the 1:1:1 multispecies biofilm (Figure 4A). The *S. gordonii* and *S. mutans* mono-species biofilms had the lowest yield strains at 0.0153 and 0.0146 Pa, respectively (Figure 4B). The 1:1:0.1 multispecies biofilm had a significantly higher yield strain compared to the Streptococci mono-species biofilms. All biofilms containing *C. albicans* had similar strain rates from 0.0166 to 0.0171 Pa. An overall increase in the yield stress was seen with an increase in the concentration of *C. albicans* in the initial biofilm inoculum (7–10 Pa) (Figure 4C). The 1:1:0.1 multispecies biofilm had a yield stress most similar to *S. mutans* (4.5 Pa), with a significantly reduced stress required for mechanical yielding compared to *S. gordonii* (12.4 Pa) and *C. albicans* (45 Pa).

## 4. Discussion

In vitro models have traditionally been used to screen potential agents and determine their mechanism of action using precisely controlled experimental conditions. In developing new treatments for dental caries, chemical, microbiological, and microbial-based de- and remineralization models can be used to demonstrate the antimicrobial efficacy or the influence of a treatment on the de- and remineralization process [17,22]. The use of in vitro models has recently demonstrated the potential for novel non-invasive treatment options using preservatives and natural antimicrobial compounds [33], xylitol [26], probiotics [94,95,96,97], hydrogen peroxide [98], and nanoparticles [99,100,101]. While the discovery of these potential treatments is promising, the development of a simple, yet robust, high-throughput screening model system is needed for the rapid identification and optimization of novel treatments that can demonstrate the correction of oral microbiome dysbiosis.

Unlike previous reports, the current study aimed to evaluate the growth and suitability of a multi-species model consisting of *S. mutans*, *C. albicans*, and *S. gordonii* in a 96-well microtiter plate system for future HTS applications. The following discussion is organized in the order of the model development considerations—microbiological media and inoculum selection, growth kinetics, and in vitro cariogenic potential. Additionally, the biofilms’ mechanical stability was evaluated using a low-throughput agar plate system, which can be used to quantitatively demonstrate a structure-based mechanism of action for novel therapeutics.

### 4.1. The 1:1 v/v RPMI 1640:TSBYE 0.6% Media Allowed for Synergistic Interactions between the Microorganisms

Based on all of the biofilm metabolic activity, viability, biomass, and total microbial counts, the 1:1 *v*/*v* RPMI 1640:TSBYE 0.6% media was selected as a good media for a robust in vitro, defined consortia multispecies biofilm, with 1:1 *v*/*v* RPMI 1640:THBYE 0.3% being considered an appropriate alternative. While these two media have similar overall mixtures of proteins, carbohydrates, sugar, and vitamins, the concentrations within each media and the average pH of the two media are different, which may explain the variation in biofilm growth (Table S1). 1:1 *v*/*v* RPMI 1640:TSBYE 0.6% also provided support for all mono-species biofilms, though to a lesser extent for *C. albicans*. RPMI-1640 containing media have also been shown to induce germ tube formation in *C. albicans*, which marks the onset of hyphal growth and increased pathogenicity [65], and it has been compared in a previous dual-species *S. gordonii* + *C. albicans* model with more complicated basal medium mucin artificial saliva [30].

Following the selection of the 1:1 *v*/*v* RPMI 1640:TSBYE 0.6% as the microbiological media, we tested various inoculum concentrations on the growth of the dual- and tri-species biofilms. The dual species biofilms were assessed to determine specific synergistic or antagonistic effects between each streptococcus species and *C. albicans* and between the two Streptococci. Both the *S. gordonii: C. albicans* and *S. mutans: C. albicans* biofilms had a higher metabolic activity and overall total microbial counts than the mono-species biofilms [102]. This supports the previously reported commensal relationships between *C. albicans* and *S. mutans* [32,35,55,73]. In brief, the symbiotic relationship between *S. mutans* and *C. albicans* has been indicated as an important factor in the ability of *C. albicans* to colonize the tooth hard surface, the production of ECM components, and the pathogenesis of early childhood caries (ECC) [55,103]. Previous studies have also shown that interactions exist between *S. gordonii* and *C. albicans* [31,56], and that these interactions enhance *C. albicans’* hyphal development and therefore pathogenicity in vitro [104,105]. The biofilm metabolic activity and total microbial counts for the *S. gordonii: S. mutans* dual species biofilm were concentration dependent, which is supported by the observation that *S. gordonii* and *S. mutans* are competitive [63,106], with *S. mutans* dominating in the caries biofilm in vivo [107]. Finally, we see an overall balance in the metabolic activity and total microbial counts for the tri-species biofilm, which supports the overall synergistic and balancing effect these microorganisms have in vitro.

### 4.2. A Stable Phase of Biofilm Growth for Therapeutic Screening was Identified through Biofilm Kinetic Studies

In order for a new biofilm model to be useful for the high-throughput screening of novel anti-biofilm therapies, there needs to be a stable phase of growth during which therapies can be tested without dynamic growth effects skewing the data. It is important to note that biofilm-prevention therapies can be tested under dynamic growth conditions [76]. Biofilm growth over 48 h was evaluated using metabolic activity, viability, biomass, and pH assays.

Overall, we observed that all biofilms had growth curves that were representative of a closed model system with an initial growth phase, followed by stagnant growth, before a decline is seen as nutrients become scarce [25]. Since fresh media was added at the 24-h time point, the decline in the biofilm viability is controlled, and fluctuations in growth between 24 and 48 h are minimized. The *S. gordonii* mono-species biofilm had the most fluctuations in the metabolic activity and viability. This biofilm also had the least amount of biomass, which may indicate that less extracellular polymeric substances were generated to form the protective ECM, leading to a bigger environmental impact on the bacteria within the biofilm.

### 4.3. Multispecies Biofilms are More Stable than Mono-Species Biofilms

Overall, we observed, through the metabolic activity, biomass, and viable colony enumeration, that the biofilm growth was stable between 10 and 48 h for all of the multispecies biofilms. This is a longer stable phase than the one observed for any of the mono-species biofilms. Again, the synergistic and commensal relationships between the microorganisms in this model likely contribute to the stable growth of these biofilms. This 38-h stable phase allows for the extensive testing of therapeutics in vitro. In many biofilm studies involving 96 well plate systems, anti-biofilm testing is restricted to maximum exposure times of 24 h due to the decline in nutrients over time for the closed system [25]. Overall, the crystal violet biomass staining of the multispecies models also showed a rapid increase in the biomass until 10 h. Between 14 and 24 h, the multispecies biofilm biomass was more variable before declining slightly after 24 h. A slight decline in the biomass is noted between 20 and 24 h for the *S. mutans, S. gordonii*, and 1:1:0.1 biofilms. The model developed here, therefore aligns with previously established protocols and supports the appropriateness of this model system for the high-throughput screening of anti-biofilm therapeutics [108,109,110].

### 4.4. Biofilm pH was Estimated in a 96-Well Plate System and Confirmed Acidic Microenvironment

A primary concern with caries-associated biofilms is the generation of an acidic microenvironment as bacteria, such as *S. mutans*, metabolize sugars, glucans, and other carbohydrates [111]. *S. mutans*, along with other bacteria, acidify these carbon sources and change the etiology of the caries biofilm, shifting the microbial composition to favor acidogenic and acid-tolerating species [112,113]. The acidic microenvironment of caries-associated biofilms attached at the tooth pellicle is the primary concern for the erosion of dental tissues, as saliva provides a neutralizing buffer throughout the oral cavity overall [58]. Studies testing the dissolution of hard tooth surfaces, including enamel and dentin, have found that the ‘critical pH’ for enamel dissolution is 5.5 [1,114], with the demineralization rate increasing as the pH decreases [115].

The microenvironment pH is also an important factor in developing novel therapeutics for anti-caries treatment. New therapies must be stable or controllable across a range of pHs, from the overall neutral environment of the oral cavity to the various acidities that form within the biofilm microenvironments. While other studies of pH effects on caries models have used large scale physical methods, including electrodes, microradiography, nano-indentation, and electron microscopy, the determination of calcium and phosphate release [115,116,117], very few have focused on the dynamic change in pH as the biofilm develops and grows. An acid sensitive dye was therefore tested and used as the primary method of detecting changes in the biofilm supernatant microenvironment pH. Bromocresol green changes from yellow to blue as the pH increases from 3.8 to 5.4 [118], which allows for the detection of enamel dissolution levels within the in vitro model system. Bromocresol green has been used as a pH indicator in various growth mediums for microorganisms and titrations, as well as for clinical applications to detect possible cases of renal failure or liver disease [88]. An acid sensitive dye was selected, rather than a pH micro-electrode, in order to rapidly determine the biofilm supernatant pH in a 96-well format applicable to HTS systems. One limitation of BCG is the dependence on absorbance measurements to estimate the pH of the biofilm microenvironment, which can be impacted by the concentration and type of microorganisms present, as we have seen with the *C. albicans* and 1:1:1 multispecies biofilms (Figure S1). Over time, for all biofilms expect the 1:1:1 biofilm, the pH decreased as the biofilm grew and aged. For all of the tested biofilm models, the final pH was below 5.0, which demonstrates the potential of these biofilms to represent the acidic action of caries-associated biofilms in vivo.

### 4.5. Multispecies Mechanical Biofilm Properties Correspond to Properties of the Prevalent Species

The implementation of advanced mechanical rheological techniques also demonstrated the combined effect of the microorganisms on the mechanical properties of the multispecies biofilm. The increase in the elastic modulus, G’, as a function of the *C. albicans* concentration in the multispecies biofilms, indicates that the presence of *C. albicans* increases the intermolecular and cohesive forces within the biofilm matrix and therefore the rigidity, despite the significant EPS production from *S. mutans* [59]. Previous studies of the *C. albicans* mechanical properties have shown that an increased hyphal development, which indicates in vitro pathogenicity, leads to increased elasticity [119], which is here related to the oscillatory measurements through Poisson’s ratio μ [120]. The measurements of the yield strain, or the deformation that can occur before mechanical failure begins, indicate that the *C. albicans* mono-species biofilm and all three multispecies biofilms are better able to handle the strain of deformation than the two Streptococci strains can. Finally, the yield stress measurements indicate that the strength of the biofilm is significantly higher for the mono-species *C. albicans* biofilm compared to all other biofilms [121]. Other research groups have found that multispecies biofilms are better able to withstand compressive forces than their counterpart mono-species biofilm can [122], while our results demonstrate that the multispecies biofilms are more vulnerable to oscillatory forces than the *C. albicans* mono-species biofilm are.

## 5. Conclusions

In summary, we report here, for the first time, an in vitro model in an efficient 96-well microtiter plate system, including commonly isolated species from dental caries: *S. gordonii, S. mutans*, and *C. albicans*. We found 1:1 *v*/*v* RPMI 1640:TSBYE 0.6% to be the best media for the development of the 1:1:0.1 multi-kingdom, multispecies model during a 24-h period. We compared the use of the common metabolic activity assay, XTT proliferation, with the newer PrestoBlue assay, when used with this model; we demonstrated the inappropriateness of using an XTT reduction assay with this model [93]. The results also indicate the ability of this model to be used for the screening of anti-caries therapies over a 48-h period, with a good stability in the biofilm metabolic activity and biomass. We have also demonstrated the use of pH sensitive dyes, including Bromocresol green, for determining the cariogenic potential, or acidity, of the biofilm microenvironment. Finally, we have shown that the mechanical properties are different for the different microorganisms, with the multispecies biofilms reflecting these differences. These characterization methods provide strong evidence that this model can be used for HTS assays. It is important to note that this model was designed for the efficient high throughput screening of therapeutics; and while the model contains inter-kingdom relationships, commensal bacteria, and acidogenic bacteria, there are inherent limitations in the ability of these three microorganisms to mimic the complex biofilms that develop in the oral cavity. In future, the 96-well plate model could be used for the high-throughput screening of novel anti-caries treatments, while the rheology model could be used to better understand the effects that novel treatments have on the mechanical stability of these oral caries-associated biofilms.

## Supplementary Materials

The following are available online at https://www.mdpi.com/2571-5135/8/2/14/s1, Table S1: Microbiological media compositions and media pH evaluated for mono- and multispecies biofilm growth, Figure S1: Use of Bromocresol Green dye for high-throughput determination of biofilm microenvironment pH, Table S2: Biofilm growth in various media data from three independent replicates, Table S3: Biofilm growth over 48 h period in 1:1 *v*/*v* RPMI 1640:TSBYE 0.6% data from two independent replicates, Figure S2: Oscillatory Frequency and Strain Sweeps.

## References

[1] Marsh P.D. (2006). Dental plaque as a biofilm and a microbial community—Implications for health and disease. BMC Oral Health.

[2] Selwitz R.H., Ismail A.I., Pitts N.B. (2007). Dental caries. Lancet.

[3] Dewhirst F.E., Chen T., Izard J., Paster B.J., Tanner A.C., Yu W.H., Lakshmanan A., Wade W.G. (2010). The human oral microbiome. J. Bacteriol..

[4] Ghannoum M.A., Jurevic R.J., Mukherjee P.K., Cui F., Sikaroodi M., Naqvi A., Gillevet P.M. (2010). Characterization of the oral fungal microbiome (mycobiome) in healthy individuals. PloS Pathog..

[5] Do T., Devine D., Marsh P.D. (2013). Oral biofilms: Molecular analysis, challenges, and future prospects in dental diagnostics. Clin. Cosmet. Investig. Dent..

[6] Munson M.A., Banerjee A., Watson T.F., Wade W.G. (2004). Molecular analysis of the microflora associated with dental caries. J. Clin. Microbiol..

[7] Verma D., Garg P.K., Dubey A.K. (2018). Insights into the human oral microbiome. Arch. Microbiol..

[8] Marsh P.D., Moter A., Devine D.A. (2011). Dental plaque biofilms: Communities, conflict, and control. Periodontology 2000.

[9] Flemmig T.F., Beikler T. (2011). Control of oral biofilms. Periodontology 2000.

[10] Metwalli K.H., Khan S.A., Krom B.P., Jabra-Rizk M.A. (2013). Streptococcus mutans, Candida albicans, and the human mouth: A sticky situation. PLoS Pathog..

[11] Department of Health and Human Services (2003). Oral Health Care Drug Products for Over-the-Counter Human Use; Antigingivitis/Antiplaque Drug Products; Establishment of a Monograph; Proposed Rules. Fed. Regist..

[12] Wong A., Subar P.E., Young D.A. (2017). Dental Caries: An Update on Dental Trends and Therapy. Adv. Pediatr..

[13] Kassebaum N.J., Smith A.G.C., Bernabé E., Fleming T.D., Reynolds A.E., Vos T., Murray C.J.L., Marcenes W., Collaborators G.O.H. (2017). Global, Regional, and National Prevalence, Incidence, and Disability-Adjusted Life Years for Oral Conditions for 195 Countries, 1990–2015: A Systematic Analysis for the Global Burden of Diseases, Injuries, and Risk Factors. J. Dent. Res..

[14] Frencken J.E., Sharma P., Stenhouse L., Green D., Laverty D., Dietrich T. (2017). Global epidemiology of dental caries and severe periodontitis—A comprehensive review. J. Clin. Periodontol..

[15] Wierichs R.J., Meyer-Lueckel H. (2015). Systematic review on noninvasive treatment of root caries lesions. J. Dent. Res..

[16] Braga A., Pires J., Magalhães A.C. (2017). Commercial antimicrobials mouthrinses on caries and periodontitis-related biofilm control: A review of literature. Braz. Dent. Sci..

[17] Fernandez C.E., Tenuta L.M., Cury J.A. (2016). Validation of a Cariogenic Biofilm Model to Evaluate the Effect of Fluoride on Enamel and Root Dentine Demineralization. PLoS ONE.

[18] Marsh P.D. (2012). Contemporary perspective on plaque control. Br. Dent. J..

[19] Gostemeyer G., Kohls A., Paris S., Schwendicke F. (2018). Root caries prevention via sodium fluoride, chlorhexidine and silver diamine fluoride in vitro. Odontology.

[20] Söderström U., Johansson I., Sunnegårdh-Grönberg K. (2014). A retrospective analysis of caries treatment and development in relation to assessed caries risk in an adult population in Sweden. BMC Oral Health.

[21] Maske T.T., van de Sande F.H., Arthur R.A., Huysmans M., Cenci M.S. (2017). In vitro biofilm models to study dental caries: A systematic review. Biofouling.

[22] Xuelin H., Qiang G., Biao R., Yuqing L., Xuedong Z., Xuedong Z. (2016). Models in Caries Research. Dental Caries: Principles and Management.

[23] Yu O.Y., Zhao I.S., Mei M.L., Lo E.C.-M., Chu C.-H. (2017). Dental Biofilm and Laboratory Microbial Culture Models for Cariology Research. Dent. J..

[24] Darrene L.N., Cecile B. (2016). Experimental Models of Oral Biofilms Developed on Inert Substrates: A Review of the Literature. Biomed. Res. Int..

[25] Sim C.P.C., Dashper S., Reynolds E.C. (2016). Oral microbial biofilm models and their application to the testing of anticariogenic agents. J. Dent..

[26] Salli K.M., Forssten S.D., Lahtinen S.J., Ouwehand A.C. (2016). Influence of sucrose and xylitol on an early Streptococcus mutans biofilm in a dental simulator. Arch. Oral Biol..

[27] Zhou J., Horev B., Hwang G., Klein M.I., Koo H., Benoit D.S. (2016). Characterizaiton and optimization of pH-responsive polymer nanoparticles for drug delivery to oral biofilms. J. Mater. Chem. B.

[28] Prasanth M. (2011). Antimicrobial efficacy of different toothpastes and mouthrinses: An in vitro study. Dent. Res. J..

[29] Standar K., Kreikemeyer B., Redanz S., Munter W.L., Laue M., Podbielski A. (2010). Setup of an in vitro test system for basic studies on biofilm behavior of mixed-species cultures with dental and periodontal pathogens. PLoS ONE.

[30] Montelongo-Jauregui D., Srinivasan A., Ramasubramanian A.K., Lopez-Ribot J.L. (2016). An In Vitro Model for Oral Mixed Biofilms of *Candida albicans* and *Streptococcus gordonii* in Synthetic Saliva. Front. Microbiol..

[31] Ricker A., Vickerman M., Dongari-Bagtzoglou A. (2014). *Streptococcus gordonii* glucosyltransferase promotes biofilm interactions with Candida albicans. J. Oral Microbiol..

[32] Ellepola K., Liu Y., Cao T., Koo H., Seneviratne C.J. (2017). Bacterial GtfB Augments *Candida albicans* Accumulation in Cross-Kingdom Biofilms. J. Dent. Res..

[33] Cieplik F., Kara E., Muehler D., Enax J., Hiller K.A., Maisch T., Buchalla W. (2018). Antimicrobial efficacy of alternative compounds for use in oral care toward biofilms from caries-associated bacteria in vitro. Microbiologyopen.

[34] Cieplik F., Wimmer F., Muehler D., Thurnheer T., Belibasakis G., Hiller K., Maisch T., Buchalla W. (2018). Phenalen-1-One-Mediated Antimicrobial Photodynamic Therapy and Chlorhexidine Applied to a Novel Caries Biofilm Model. Caries Res..

[35] Kim D., Sengupta A., Niepa T.H., Lee B.H., Weljie A., Freitas-Blanco V.S., Murata R.M., Stebe K.J., Lee D., Koo H. (2017). *Candida albicans* stimulates *Streptococcus mutans* microcolony development via cross-kingdom biofilm-derived metabolites. Sci. Rep..

[36] Shapiro S., Giertsen E., Guggenheim B. (2002). An in vitro oral biofilm model for comparing the efficacy of antimicrobial mouthrinses. Caries Res..

[37] Guggenheim B., Guggenheim M., Gmur R., Giertsen E., Thurnheer T. (2004). Application of the Zurich biofilm model to problems of cariology. Caries Res..

[38] Xiao J., Klein M.I., Falsetta M.L., Lu B., Delahunty C.M., Yates J.R., Heydorn A., Koo H. (2012). The exopolysaccharide matrix modulates the interaction between 3D architecture and virulence of a mixed-species oral biofilm. PLoS Pathog..

[39] Thurnheer T., Bostanci N., Belibasakis G.N. (2015). Microbial dynamics during conversion from supragingival to subgingival biofilms in an in vitro model. Mol. Oral Microbiol..

[40] Sherry L., Lappin G., O’Donnell L.E., Millhouse E., Millington O.R., Bradshaw D.J., Axe A.S., Williams C., Nile C.J., Ramage G. (2016). Viable Compositional Analysis of an Eleven Species Oral Polymicrobial Biofilm. Front. Microbiol..

[41] Guggenheim B., Giertsen E., Schupbach P., Shapiro S. (2001). Validation of an in vitro biofilm model of supragingival plaque. J. Dent. Res..

[42] Exterkate R.A., Crielaard W., Ten Cate J.M. (2010). Different response to amine fluoride by *Streptococcus mutans* and polymicrobial biofilms in a novel high-throughput active attachment model. Caries Res..

[43] Bradshaw D.J., Marsh P.D. (1998). Analysis of pH-Driven Disruption of oral microbial communities in vitro. Caries Res..

[44] Shu M., Wong L., Miller J.H., Sissions C.H. (2000). Development of multi-species consorita biofilms of oral bacteria as an enamel and root caries model system. Arch. Oral Biol..

[45] Filoche S.K., Anderson S.A., Sissions C.H. (2004). Biofilm growth of Lactobacillus species is promoted by Actinomyces species and Streptococcus mutans. Oral Microbiol. Immunol..

[46] Wong L., Sissions C.H. (2001). A comparison of human dental plaque microcosm biofilms grown in an undefined medium and a chemically defined artificial saliva. Arch. Oral Biol..

[47] Foster J.S., Kolenbrander P.E. (2004). Development of a multispecies oral bacterial community in a saliva-conditioned flow cell. Appl. Environ. Microbiol..

[48] Hope C.K., Bakht K., Burnside G., Martin G.C., Burnett G., de Josselin de Jong E., Higham S.M. (2012). Reducing the variability between constant-depth film fermenter experiments when modelling oral biofilm. J. Appl. Microbiol..

[49] Mayr L.M., Bojanic D. (2009). Novel trends in high-throughput screening. Curr. Opin. Pharm..

[50] Kolenbrander P.E., Palmer R.J., Periasamy S., Jakubovics N.S. (2010). Oral multispecies biofilm development and the key role of cell-cell distance. Nat. Rev. Microbiol..

[51] Sanz M., Beighton D., Curtis M.A., Cury J.A., Dige I., Dommisch H., Ellwood R., Giacaman R.A., Herrera D., Herzberg M.C. (2017). Role of microbial biofilms in the maintenance of oral health and in the development of dental caries and periodontal diseases. Consensus report of group 1 of the Joint EFP/ORCA workshop on the boundaries between caries and periodontal disease. J. Clin. Periodontol..

[52] Hoare A., Marsh P.D., Diaz P.I. (2017). Ecological Therapeutic Opportunities for Oral Diseases. Microbiol. Spectr..

[53] United States Pharmacopeia and National Formulary (USP 40-NF 35) (2017). <61> Microbiological Examination of Nonsterile Products: Microbial Enumeration Tests.

[54] Trigo-Gutierrez J.K., Sanita P.V., Tedesco A.C., Pavarina A.C., Mima E.G.O. (2018). Effect *of Chloroaluminium phthalocyanine* in cationic nanoemulsion on photoinactivation of multispecies biofilm. Photodiagnosis Photodyn.

[55] Falsetta M.L., Klein M.I., Colonne P.M., Scott-Anne K., Gregoire S., Pai C.-H., Gonzalez-Begne M., Watson G., Krysan D.J., Bowen W.H. (2014). Symbiotic Relationship between *Streptococcus mutans* and *Candida albicans* Synergizes Virulence of Plaque Biofilms in vivo. Infect. Immun..

[56] Diaz P.I., Xie Z., Sobue T., Thompson A., Biyikoglu B., Ricker A., Ikonomou L., Dongari-Bagtzoglou A. (2012). Synergistic interaction between *Candida albicans* and commensal oral streptococci in a novel in vitro mucosal model. Infect. Immun..

[57] De Carvalho F.G., Silva D.S., Hebling J., Spolidorio L.C., Spolidorio D.M. (2006). Presence of mutans streptococci and *Candida* spp. in dental plaque/dentine of carious teeth and early childhood caries. Arch. Oral Biol..

[58] Koo H., Falsetta M.L., Klein M.I. (2013). The exopolysaccharide matrix: A virulence determinant of cariogenic biofilm. J. Dent. Res..

[59] Klein M.I., Hwang G., Santos P.H., Campanella O.H., Koo H. (2015). Streptococcus mutans-derived extracellular matrix in cariogenic oral biofilms. Front. Cell. Infect. Microbiol..

[60] Hajishengallis E., Parsaei Y., Klein M.I., Koo H. (2017). Advances in the microbial etiology and pathogenesis of early childhood caries. Mol. Oral Microbiol..

[61] Ajdic D., McShan W.M., McLaughlin R.E., Savic G., Chang J., Carson M.B., Primeaux C., Tian R., Kenton S., Jia H. (2002). Genome sequence of *Streptococcus mutans* UA159, a cariogenic dental pathogen. PNAS.

[62] Burne R.A. (2018). Getting to Know “The Known Unknowns”: Heterogeneity in the Oral Microbiome. Adv. Dent. Res..

[63] Huang R., Li M., Gregory R.L. (2011). Bacterial interactions in dental biofilm. Virulence.

[64] Thein Z.M., Seneviratne C.J., Samaranayake Y.H., Samaranayake L.P. (2009). Community lifestyle of Candida in mixed biofilms: A mini review. Mycoses.

[65] Ellepola A.N., Joseph B.J., Khan Z.U. (2012). Effects of subtherapeutic concentrations of chlorhexidine gluconate on germ tube formation of oral Candida. Med. Princ. Pract..

[66] Darwazeh A.M.G., Darwazeh T.A. (2014). What Makes Oral Candidiasis Recurrent Infection? A Clinical View. J. Mycol..

[67] Byadarahally Raju S., Rajappa S. (2011). Isolation and identification of Candida from the oral cavity. ISRN Dent..

[68] Marsh P.D. (2010). Controlling the oral biofilm with antimicrobials. J. Dent..

[69] Ramsey M.M., Rumbaugh K.P., Whiteley M. (2011). Metabolite cross-feeding enhances virulence in a model polymicrobial infection. PLoS Pathog..

[70] Stacy A., Abraham N., Jorth P., Whiteley M. (2016). Microbial Community Composition Impacts Pathogen Iron Availability during Polymicrobial Infection. PLoS Pathog..

[71] Pierce C.G., Uppuluri P., Lopez-Ribot J.L., Gupta V.K. (2013). A Method for the Formation of Candida Biofilms in 96 Well Microtiter Plates and Its Application to Antifungal Susceptibility Testing. Laboratory Protocols in Fungal Biology: Current Methods in Fungal Biology.

[72] Krom B.P., Willems H.M.E., Caderone R., Cihlar R. (2016). In Vitro Models for Candida Biofilm Development. Candida Species: Methods and Protcols.

[73] Sztajer H., Szafranski S.P., Tomasch J., Reck M., Nimtz M., Rohde M., Wagner-Dobler I. (2014). Cross-feeding and interkingdom communication in dual-species biofilms of *Streptococcus mutans* and *Candida albicans*. ISME J..

[74] Cavazana T.P., Pessan J.P., Hosida T.Y., Monteiro D.R., Botazzo Delbem A.C. (2018). pH changes of mixed biofilms of *Streptococcus mutans* and *Candida albicans* after exposure to sucrose solutions in vitro. Arch. Oral Biol..

[75] Aymanns S., Mauerer S., van Zandbergen G., Wolz C., Spellerberg B. (2011). High-level fluorescence labelling of gram-positive pathogens. PLoS ONE.

[76] Bahamondez-Canas T., Smyth H.D.C. (2018). Influence of Excipients on the Antimicrobial Activity of Tobramycin Against *Pseudomonas aeruginosa* Biofilms. Pharm. Res..

[77] Molecular Probes Inc. (2010). PrestoBlue Cell Viability Reagent Protocol.

[78] Sarker S.D., Nahar L., Kumarasamy Y. (2007). Microtitre plate-based antibacterial assay incorporating resazurin as an indicator of cell growth, and its application in the in vitro antibacterial screening of phytochemicals. Methods.

[79] Van den Driessche F., Rigole P., Brackman G., Coenye T. (2014). Optimization of resazurin-based viability staining for quantification of microbial biofilms. J. Microbiol. Methods.

[80] Molecular Probes Inc. (2011). Application Note: Processing Absorbance Data Obtained Using PrestoBlue Viability Reagent.

[81] Bandara H.M., Nguyen D., Mogarala S., Osinski M., Smyth H.D. (2015). Magnetic fields suppress *Pseudomonas aeruginosa* biofilms and enhance ciprofloxacin activity. Biofouling.

[82] Azeredo J., Azevedo N.F., Briandet R., Cerca N., Coenye T., Costa A.R., Desvaux M.L., Bonaventura G.D., Heébraud M., Jaglic Z. (2017). Critical review on biofilm methods. Crit. Rev. Microbiol..

[83] Gold O.G., Jordan H.V., Houte J.V. (1974). Identification of Streptococcus mutans colonies by mannitol-dependent tetrazolium reduction. Arch. Oral Biol..

[84] Momeni S.S., Patrick P., Wiener H.W., Cutter G.R., Ruby J.D., Cheon K., Whiddon J., Moser S.A., Childers N.K. (2014). Mutans streptococci enumeration and genotype selection using different bacitracin-containing media. J. Microbiol. Methods.

[85] United States Pharmacopeia and National Formulary (USP 40-NF 35) (2017). <62> Microbiological Examination of Nonsterile Products: Test for Specified Microorganisms.

[86] Villhauer A.L., Lynch D.J., Drake D.R. (2017). Improved method for rapid and accurate isolation and identification of *Streptococcus mutans* and *Streptococcus sobrinus* from human plaque samples. J. Microbiol. Methods.

[87] Lindsay A.K., Morales D.K., Liu Z., Grahl N., Zhang A., Willger S.D., Myers L.C., Hogan D.A. (2014). Analysis of *Candida albicans* mutants defective in the Cdk8 module of mediator reveal links between metabolism and biofilm formation. PLoS Genet..

[88] Kim M.J., Jung S.W., Park H.R., Lee S.J. (2012). Selection of an optimum pH-indicator for developing lactic acid bacteria-based time–temperature integrators (TTI). J. Food Eng..

[89] Zarei K., Atabati M., Abdinasab E. (2009). Spectrophotometric Determination of Conditional Acidity Constant of Some Sulfonephthalein Dyes as a Function of Anionic, Neutral and Cationic Surfactants Concentrations Using Rank Annihilation Factor Analysis. Eurasian J. Anal. Chem..

[90] Beckett A.H., Stenlake J.B. (1988). Practical Pharmaceutical Chemistry: Part. II.

[91] Kovach K., Davis-Fields M., Irie Y., Jain K., Doorwar S., Vuong K., Dhamani N., Mohanty K., Touhami A., Gordon V.D. (2017). Evolutionary adaptations of biofilms infecting cystic fibrosis lungs promote mechanical toughness by adjusting polysaccharide production. NPJ Biofilms Microbiomes.

[92] R Core Team (2013). R: A Language and Environment for Statistical Computing.

[93] Lall N., Henley-Smith C.J., De Canha M.N., Oosthuizen C.B., Berrington D. (2013). Viability Reagent, PrestoBlue, in Comparison with Other Available Reagents, Utilized in Cytotoxicity and Antimicrobial Assays. Int. J. Microbiol..

[94] Baca-Castanon M.L., De la Garza-Ramos M.A., Alcazar-Pizana A.G., Grondin Y., Coronado-Mendoza A., Sanchez-Najera R.I., Cardenas-Estrada E., Medina-De la Garza C.E., Escamilla-Garcia E. (2015). Antimicrobial Effect of *Lactobacillus reuteri* on Cariogenic Bacteria *Streptococcus gordonii*, *Streptococcus mutans*, and Periodontal Diseases *Actinomyces naeslundii* and *Tannerella forsythia*. Probiotics Antimicrob. Proteins.

[95] Hillman J.D. (2006). Compositions and Methods for the Maintenance of Oral Health. United States Patent Application.

[96] Gruner D., Paris S., Schwendicke F. (2016). Probiotics for managing caries and periodontitis: Systematic review and meta-analysis. J. Dent..

[97] Tan Y., Leonhard M., Moser D., Ma S., Schneider-Stickler B. (2017). Inhibitory effect of probiotic lactobacilli supernatants on single and mixed non-albicans Candida species biofilm. Arch. Oral Biol..

[98] Gao L., Liu Y., Kim D., Li Y., Hwang G., Naha P.C., Cormode D.P., Koo H. (2016). Nanocatalysts promote *Streptococcus mutans* biofilm matrix degradation and enhance bacterial killing to suppress dental caries in vivo. Biomaterials.

[99] Alas G., Pagano R.E., Nguyen J.Q., Bandara H.M.H.N., Ivanov S.A., Smolyakov G.A., Huber D.L., Smyth H.D.C., Osiński M. (2017). Effects of iron-oxide nanoparticles and magnetic fields on oral biofilms. Proc. SPIE.

[100] Pelgrift R.Y., Friedman A.J. (2013). Nanotechnology as a therapeutic tool to combat microbial resistance. Adv. Drug Deliv. Rev..

[101] Rudramurthy G.R., Swamy M.K., Sinniah U.R., Ghasemzadeh A. (2016). Nanoparticles: Alternatives Against Drug-Resistant Pathogenic Microbes. Molecules.

[102] Cavalcanti Y.W., Morse D.J., da Silva W.J., Del-Bel-Cury A.A., Wei X., Wilson M., Milward P., Lewis M., Bradshaw D., Williams D.W. (2015). Virulence and pathogenicity of *Candida albicans* is enhanced in biofilms containing oral bacteria. Biofouling.

[103] Koo H., Bowen W.H. (2014). *Candida albicans* and *Streptococcus mutans*: A potential synergistic alliance to cause virulent tooth decay in children. Future Microbiol..

[104] Bamford C.V., d’Mello A., Nobbs A.H., Dutton L.C., Vickerman M.M., Jenkinson H.F. (2009). *Streptococcus gordonii* modulates *Candida albicans* biofilm formation through intergeneric communication. Infect. Immun..

[105] Morales D.K., Hogan D.A. (2010). *Candida albicans* interactions with bacteria in the context of human health and disease. PLoS Pathog..

[106] Kreth J., Zhang Y., Herzberg M.C. (2008). Streptococcal antagonism in oral biofilms: Streptococcus sanguinis and Streptococcus gordonii interference with Streptococcus mutans. J. Bacteriol..

[107] Tanzer J.M., Thompson A., Sharma K., Vickerman M.M., Haase E.M., Scannapieco F.A. (2012). *Streptococcus mutans* out-competes *Streptococcus gordonii* in vivo. J. Dent. Res..

[108] McBain A.J. (2009). In Vitro Biofilm Models. Advances in Applied Microbiology.

[109] Klodzinska S.N., Priemel P.A., Rades T., Nielsen H.M. (2018). Combining diagnostic methods for antimicrobial susceptibility testing—A comparative approach. J. Microbiol. Methods.

[110] Roder H.L., Sorensen S.J., Burmolle M. (2016). Studying Bacterial Multispecies Biofilms: Where to Start?. Trends Microbiol..

[111] Yadav K., Prakash S. (2017). Dental Caries: A Microbiological Approach. J. Clin. Infect. Dis. Pract..

[112] Takahashi N., Nyvad B. (2011). The role of bacteria in the caries process: Ecological perspectives. J. Dent. Res..

[113] Klinke T., Kneist S., de Soet J.J., Kuhlisch E., Mauersberger S., Forster A., Klimm W. (2009). Acid production by oral strains of Candida albicans and lactobacilli. Caries Res..

[114] Lussi A., Schlueter N., Rakhmatullina E., Ganss C. (2011). Dental erosion—An overview with emphasis on chemical and histopathological aspects. Caries Res..

[115] Shellis R.P., Barbour M.E., Jones S.B., Addy M. (2010). Effects of pH and acid concentration on erosive dissolution of enamel, dentine, and compressed hydroxyapatite. Eur. J. Oral Sci..

[116] Barbour M.E., Parker D.M., Allen G.C., Jandt K.D. (2003). Human enamel dissolution in citric acid as a function of pH in the range 2.30 < pH < 6.30—A nanoindentation study. Eur. J. Oral Sci..

[117] Barbour M.E., Rees J.S. (2004). The laboratory assessment of enamel erosion: A review. J. Dent..

[118] Davis C.E., Sallisbury H.M. (1926). Chart of Indicators Useful for pH Measurments. Ind. Eng. Chem. Anal. Ed..

[119] Paramonova E., Krom B.P., van der Mei H.C., Busscher H.J., Sharma P.K. (2009). Hyphal content determines the compression strength of Candida albicans biofilms. Microbiology.

[120] Mezger T.G. (2014). The Rheology Handbook.

[121] Vinogradov A.M., Winston M., Rupp C.J., Stoodley P. (2004). Rheology of biofilms formed from the dental plaque pathogen *Streptococcus mutans*. Biofilms.

[122] Paramonova E., Kalmykowa O.J., van der Mei H.C., Busscher H.J., Sharma P.K. (2009). Impact of hydrodynamics on oral biofilm strength. J. Dent. Res..

