# Supplementary material for: A Multispecies Biofilm In Vitro Screening Model of Dental Caries for High-Throughput Susceptibility Testing
Source: High Throughput. 2019 May 30;8(2):14. doi: 10.3390/ht8020014 (PMC6631723; doi:10.3390/ht8020014)
Supplement: Supplementary file 1 [file high-throughput-08-00014-s001.zip › high-throughput-487318-suppl-proof/high-throughput-487318-suppl-proof.docx]

Supplementary Materials: A Multispecies Biofilm In Vitro Screening Model of Dental Caries for High-Throughput Susceptibility Testing

Lara A. Heersema ^1^ and Hugh D.C. Smyth

**Figure S1.** Use of Bromocresol Green dye for high-throughput determination of biofilm microenvironment pH. (**A**) Absorption spectra of biofilm supernatant sans Bromocresol Green (BCG) dye. (**B**) Absorption spectra of biofilm supernatant with 20 μL BCG dye. (**C** Ratio of maximum absorption (620 nm) to isobestic point (512 nm) of biofilm supernatant sans BCG. (**D** Ratio of maximum absorption (620 nm) to isobestic point (512 nm) of biofilm supernatant with 20 μL BCG dye.

**Figure S2.** Oscillatory Frequency and Strain Sweeps of (**A**) mono- and (**B**) multispecies biofilms measured using an 8mm parallel plate rheometer at 25^o^C from two independent replicates. Filled circles represent elastic moduli. Open circles represent viscous moduli. Frequency sweeps run at 1% strain. Strain sweeps run at 3.14 rad/s.
